# Driving ferromagnetic resonance frequency of FeCoB/PZN-PT multiferroic heterostructures to Ku-band via two-step climbing: composition gradient sputtering and magnetoelectric coupling

**DOI:** 10.1038/srep07393

**Published:** 2014-12-09

**Authors:** Shandong Li, Qian Xue, Jenq-Gong Duh, Honglei Du, Jie Xu, Yong Wan, Qiang Li, Yueguang Lü

**Affiliations:** 1 College of Physics, and Key Laboratory of Photonics Materials and Technology in Universities of Shandong, Laboratory of Fiber Materials and Modern Textile, the Growing Base for State Key Laboratory, Qingdao University, Qingdao 266071, China; 2 Department of Materials Science and Engineering, National Tsing Hua University, Hsinchu 30013, Taiwan; 3 Department of Physics, School of Science, Harbin Institute of Technology, Harbin 150001, China

## Abstract

RF/microwave soft magnetic films (SMFs) are key materials for miniaturization and multifunctionalization of monolithic microwave integrated circuits (MMICs) and their components, which demand that the SMFs should have higher self-bias ferromagnetic resonance frequency *f*_FMR_, and can be fabricated in an IC compatible process. However, self-biased metallic SMFs working at X-band or higher frequency were rarely reported, even though there are urgent demands. In this paper, we report an IC compatible process with two-step superposition to prepare SMFs, where the FeCoB SMFs were deposited on (011) lead zinc niobate–lead titanate substrates using a composition gradient sputtering method. As a result, a giant magnetic anisotropy field of 1498 Oe, 1–2 orders of magnitude larger than that by conventional magnetic annealing method, and an ultrahigh *f*_FMR_ of up to 12.96 GHz reaching Ku-band, were obtained at zero magnetic bias field in the as-deposited films. These ultrahigh microwave performances can be attributed to the superposition of two effects: uniaxial stress induced by composition gradient and magnetoelectric coupling. This two-step superposition method paves a way for SMFs to surpass X-band by two-step or multi-step, where a variety of magnetic anisotropy field enhancing methods can be cumulated together to get higher ferromagnetic resonance frequency.

Nowadays integration circuit (IC) technology is developing from system-in-package towards system-on-chip, so the integration of the passive components, such as inductors and capacitors, on one chip is usually adapted in order to miniaturize the electromagnetic devices and to increase the data transmission rate^1^,^2^. Microwave soft magnetic films (SMFs) are key materials, which can effectively miniaturize and multifunctionalize the electromagnetic components and devices, such as filters, phase shifters, isolators, circulators, thin film wireless inductors, magnetic recording write heads, antennas, etc.^3^,^4^,^5^,^6^, and reduce their occupation area in MMIC boards. Modern electronic products are heading towards high density, high-frequency, frequency tunability, lightweight and low energy consumption, etc., which give rise to increasing demands for microwave SMFs. Microwave SMFs should have higher ferromagnetic resonance (FMR) frequency (*f*_FMR_) and frequency tunability, high permeability, and are to be fabricated in an IC compatible process. In order to reduce the weight and energy consumption of MMICs, researchers are trying to make magnetic devices to be clocked under no magnetic field^7^, i.e. the magnetic properties are manipulated by non-magnetic field methods, such as stress, magnetoelectric coupling, magnetoelastic coupling, etc. instead of bulky and power-consuming electromagnets. Soft magnetic ferrites are not sufficient for MMIC devices due to their lower saturation magnetization (4πM_S_) and permeability, and especially the high fabrication temperature which is incompatible with the IC process^8^. However, metallic soft magnetic films exhibit special advantages in MMIC devices since they have higher 4πM_S_ and permeability, and good compatibility with the IC fabrication process. Therefore, self-biased metallic SMFs prepared at IC compatible process have drawn an increasing attention^9^,^10^,^11^. It was reported that the metallic SMFs are able to operate at S-band (2–4 GHz) or C-band (4–8 GHz) under self-bias condition^12^,^13^. However, to the best of our knowledge, self-biased magnetic films working at Ku-band (12–18 GHz) have not been reported, even though there are urgent needs in achieving tunablility in MMICs used in radar, aircraft, satellite, portable communication products, etc.

In this paper, we choose Fe_70_Co_30_-B alloys with high 4*π*M_S_ and permeability, and demonstrate a novel method to prepare microwave SMFs at room temperature (an IC compatible process). The novel preparation method combined two magnetic anisotropy fields H_K_ together (i.e. stress induced H_K_ by composition gradient and electric field induced tunable H_K_ via magnetoelectric coupling), realizing a two-step climbing of ferromagnetic resonance frequency. As a result, a giant H_K_ of 1498 Oe, which is 1–2 orders of magnitude larger than that by conventional magnetic annealing method, and a record high *f*_FMR_ of up to 12.96 GHz, reaching Ku-band, were obtained at zero magnetic bias field and bias electric field of 8 kVcm^−1^ in the as-deposited Fe_70_Co_30_-B/lead zinc niobate–lead titanate (PZN-PT) (hereinafter referred to as FeCoB/PZN-PT) multiferroic heterostructure films. The ferromagnetic resonance frequency of the FeCoB/PZN-PT multiferroic heterostructures can be manipulated by electric field, instead of large and energy-consuming electromagnets, from 6.30 GHz to 12.96 GHz with the electric field from 0 to 8 kVcm^−1^. The net frequency shift Δ*f*_FMR_ is as high as 6.66 GHz, and the frequency tunability Δ*f*_FMR_/*f*_FMR_ is about 106%, equivalent to 832.5 MHz cm kV^−1^. This electric field manipulation of *f*_FMR_ shift has low energy consumption and lightweight, especially suitable for manufacturing tuneable MMIC devices.

The ferromagnetic resonance frequency *f*_FMR_ of SMFs can be expressed by the Kittle equation as follows,


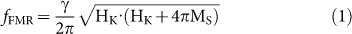
where *γ* is the gyromagnetic ratio, 4πM_S_ is the saturation magnetization. Clearly, high 4*π*M_S_ and H_K_ are needed to achieve a high *f*_FMR_ in SMFs. Previous research on achieving high *f*_FMR_ SMFs has been mostly focused on enhancing uniaxial magnetic anisotropy field H_K_ since it is relatively easier to be enhanced by 1–2 orders of magnitude, compared to saturation magnetization that is capped at 24.5 kGs at room temperature^14^,^15^. Magnetron sputtering of SMFs in *in-situ* magnetic fields and/or subsequent magnetic annealing after deposition have been widely employed for inducing a uniaxial magnetic anisotropy^16^,^17^. However, the induced uniaxial magnetic anisotropy fields are usually in the range of <50 Oe, which leads to limited ferromagnetic resonance frequency of <3 GHz for most of the metallic magnetic films^18^.

Several different approaches have been investigated for achieving high uniaxial magnetic anisotropy in SMFs, such as oblique sputtering^19^,^20^, facing-target sputtering^21^, exchange coupling^22^,^23^,^24^,^25^, and magnetoelectric coupling^26^,^27^, etc. Oblique sputtering and facing-target sputtering can generate magnetic films with H_K_ of 20–300 Oe^19^,^20^. Exchange coupling, such as antiferromagnetic/ferromagnetic exchange coupling^22^,^23^,^24^ and exchange coupling between magnetically soft and hard layers^25^, provides high H_K_ around 100–750 Oe. In our previous work, a novel composition gradient sputtering (CGS) method was applied to achieve a high uniaxial magnetic anisotropy in SMFs^28^,^29^,^30^, which dramatically increased the in-plane uniaxial magnetic anisotropy field to up to 547 Oe due to the uniaxial stress distribution induced by composition gradient. As a result, good microwave ferromagnetic properties with ferromagnetic resonance frequency over 7 GHz were obtained in composition gradient deposited magnetic films.

Multiferroic composite materials have drawn an increased amount of attention recently due to the strong magnetoelectric coupling demonstrated in multiferroic composites, which allows for electric field manipulation of magnetic properties (converse magnetoelectric effect) or magnetic field control of electric polarization (direct magnetoelectric effect)^31^,^32^,^33^,^34^. The magnetoelectric coupling in magnetic/ferroelectric multiferroic heterostructures can lead to dramatically enhanced electric-field tunable magnetic anisotropy fields approaching 750–880 Oe^35^,^36^,^37^. Based on the discussion above, it is difficult to obtain SMFs with H_K_ over 1000 Oe or *f*_FMR_ over 10 GHz at zero biased magnetic field using a single method. It is necessary to explore novel methods to enhance H_K_, and therefore to push the ferromagnetic resonance to X- or Ku-band.

## Results

### The design for enhancing the ferromagnetic resonance frequency via CGS and magnetoelecric coupling

**Figure 1:**
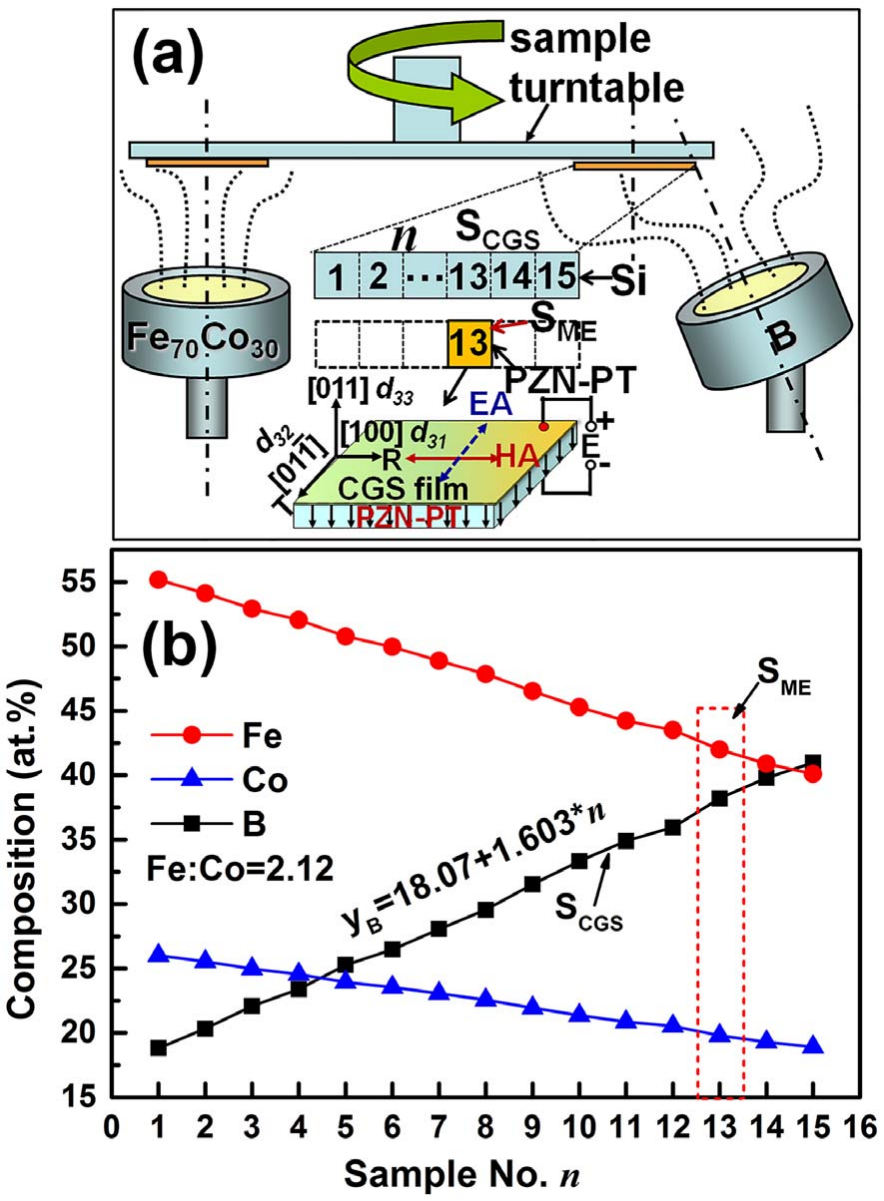
The CGS device and composition distribution. (a) The schematic drawing of composition gradient sputtering (CGS) device, and (b) the composition distribution detected by a field emission electron probe microanalyzer. The insets of Figure 1a from top to bottom shows the position distribution for CGS sample S_CGS_ and the magnetoelectric coupling sample S_ME_, and the interaction mechanism of magnetoelectric coupling between CGS film and PZN-PT. The composition distribution of S_ME_ is marked in the inset of Figure 1b using a red short-dashed box.

The FeCoB/PZN-PT multiferroic heterostructures were prepared by a composition gradient sputtering method. The detailed experimental procedures are described as follows: firstly, a (100) single crystal Si substrate with dimension of 75 mm × 5 mm × 0.5 mm was pasted on the turntable with the length direction along the radial (R) direction for optimizing fabrication condition of the CGS SMFs. The sample prepared by CGS method was named as S_CGS_. The S_CGS_ was cut into 15 segments along the length direction with equal size of 5 mm × 5 mm for microstructure and magnetic properties measurement, and the segments were successively numbered as *n* = 1 to 15 from inner to outer (see the top of Figure 1a inset). Secondly, choose an optimum position (e.g. *n* = 13 in this study), where the comprehensive soft magnetic properties are optimum, then paste a 5 mm × 5 mm (011)-cut PZN-PT single crystal substrate on this optimum position with [100] direction of PZN-PT along the R direction (i.e. [100]//R, see the middle of the Figure 1a inset ). The CGS FeCoB film was also deposited on PZN-PT substrate under the same optimum sputtering conditions for studying the electric field manipulation of ferromagnetic resonance via magnetoelectric coupling. So it was named as S_ME_. Therefore, the effects from composition gradient and magnetoelectric coupling on the microwave soft magnetic properties can be verified by the samples S_CGS_ and S_ME_, respectively.

Figure 1a shows the schematic drawing of the composition gradient sputtering method. The main target of Fe_70_Co_30_ directly faces the centre of the substrate which sits on a rotating turntable, while the doping target of B is offset radially from the sample centre. The doping gun (B target) is tilted at a certain angle towards the sample turntable. This geometrical structure ensures that the materials from the Fe_70_Co_30_ main target are distributed homogeneously across the sample, while those from B doping target will have a composition gradient distribution, i.e. B concentration increases gradually from inner to outer positions along the R orientation.

### The composition distribution and soft magnetic properties of CGS FeCoB/Si films

**Figure 2:**
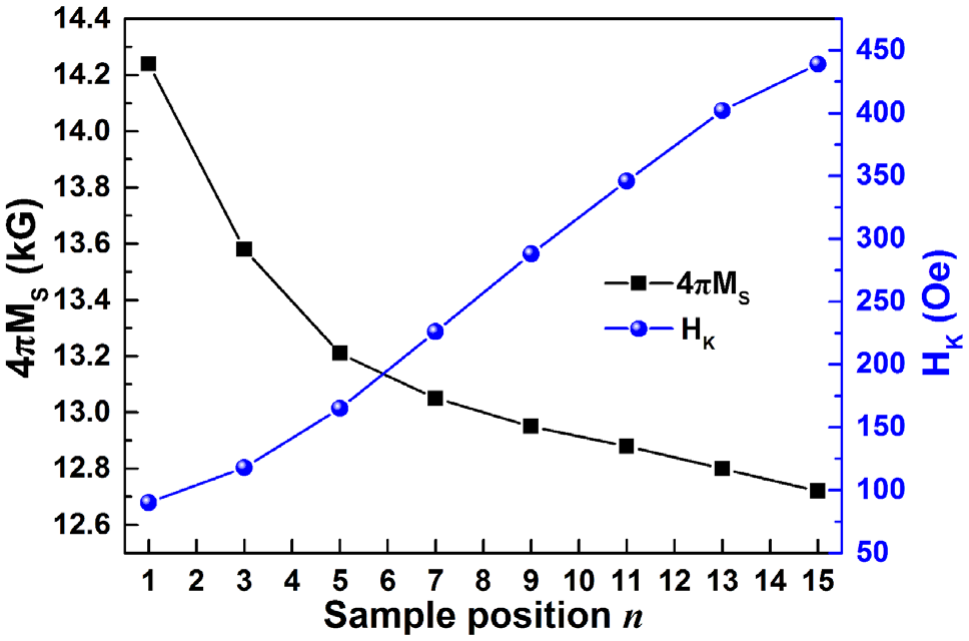
Sample position *n* dependence of 4πM_S_ and H_K_ for S_CGS_.

**Figure 3:**
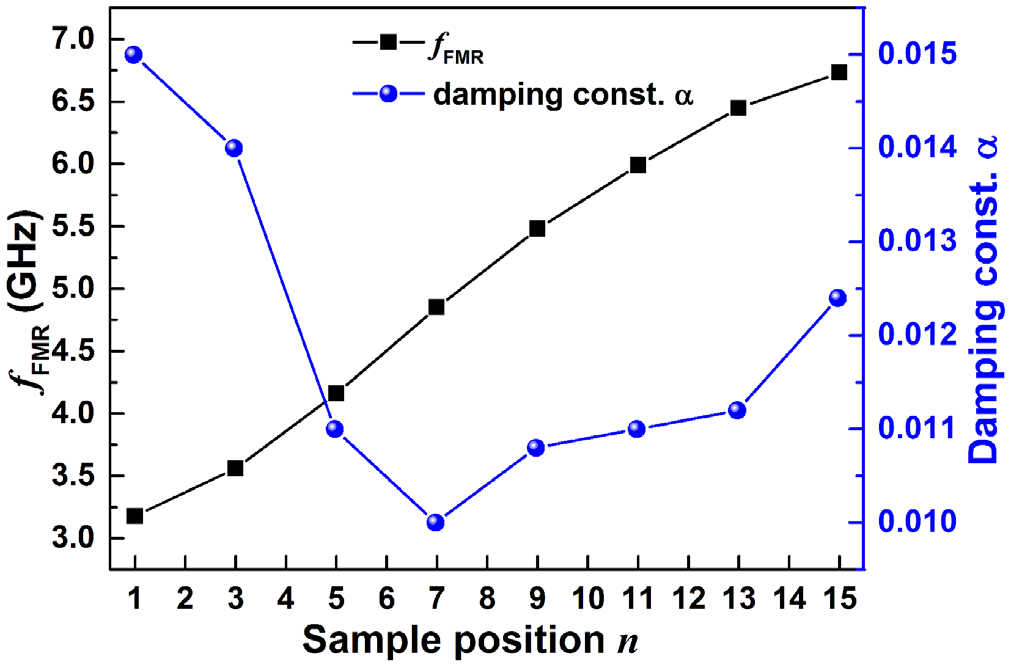
Sample position *n* dependence of *f*_FMR_ and damping constant α for S_CGS_.

The composition distribution along R direction was detected by a field emission electron probe microanalyzer (FE-EPMA). As illustrated in Figure 1b, the atomic ratio between Fe and Co remains at 2.12, indicating a homogeneous composition comparable to the Fe_70_Co_30_ target composition; while the B composition *y*_B_ increases linearly from 18.07 at.% to 42.12 at.% for the test positions from *n* = 1 to 15, undergoing a linear relation of *y*_B_ = 18.07 + 1.603*n (see Figure 1b). This composition distribution verified the design idea of using composition gradient sputtering to create a composition gradient film with linear doping. The linear doping of B element gives rise to an almost linear increase of H_K_ and a nonlinear decrease of saturation magnetization 4πM_S_. As illustrated in Figure 2, the H_K_ of CGS FeCoB films increases almost linearly from 90 to 436 Oe, while 4πM_S_ rapidly reduces from 14.24 kG (*n* = 1) to 13.21 kG (*n* = 5) at first, then decrease linearly towards 12.72 kG (*n* = 15). The H_K_ increases nearly 5 times, while 4πM_S_ reduces only about 10%, so the ferromagnetic resonance frequency is dominated by H_K_. As expected, the sample position *n* dependence of *f*_FMR_, shown in Figure 3, demonstrates almost the same trend in *f*_FMR_ with that in H_K_ (shown in Figure 2). With the increase of *n*, *f*_FMR_ increases from 3.18 to 6.73 GHz with increment of 3.55 GHz, equivalent to an increase ratio of 212%. The damping constant α of the CGS FeCoB/Si samples, shown in Figure 3, decreases with the increase of *n* from 1 to 7, then stays on a low platform with value of 0.011 till *n* = 13, after that α goes up, implying an increase of magnetic loss. Considering the comprehensive magnetic properties including 4πM_S_, H_K_, *f*_FMR_, and α for the segments at different *n*, we chose *n* = 13 as an optimum position to deposit magnetoelectric coupling sample on PZN-PT substrate since sample S_CGS_@*n* = 13 shows a relatively large *f*_FMR_ of 6.45 GHz, large H_K_ of 402 Oe and low α of 0.0112.

### Composition gradient induced uniaxial magnetic anisotropy in CGS films

**Figure 4:**
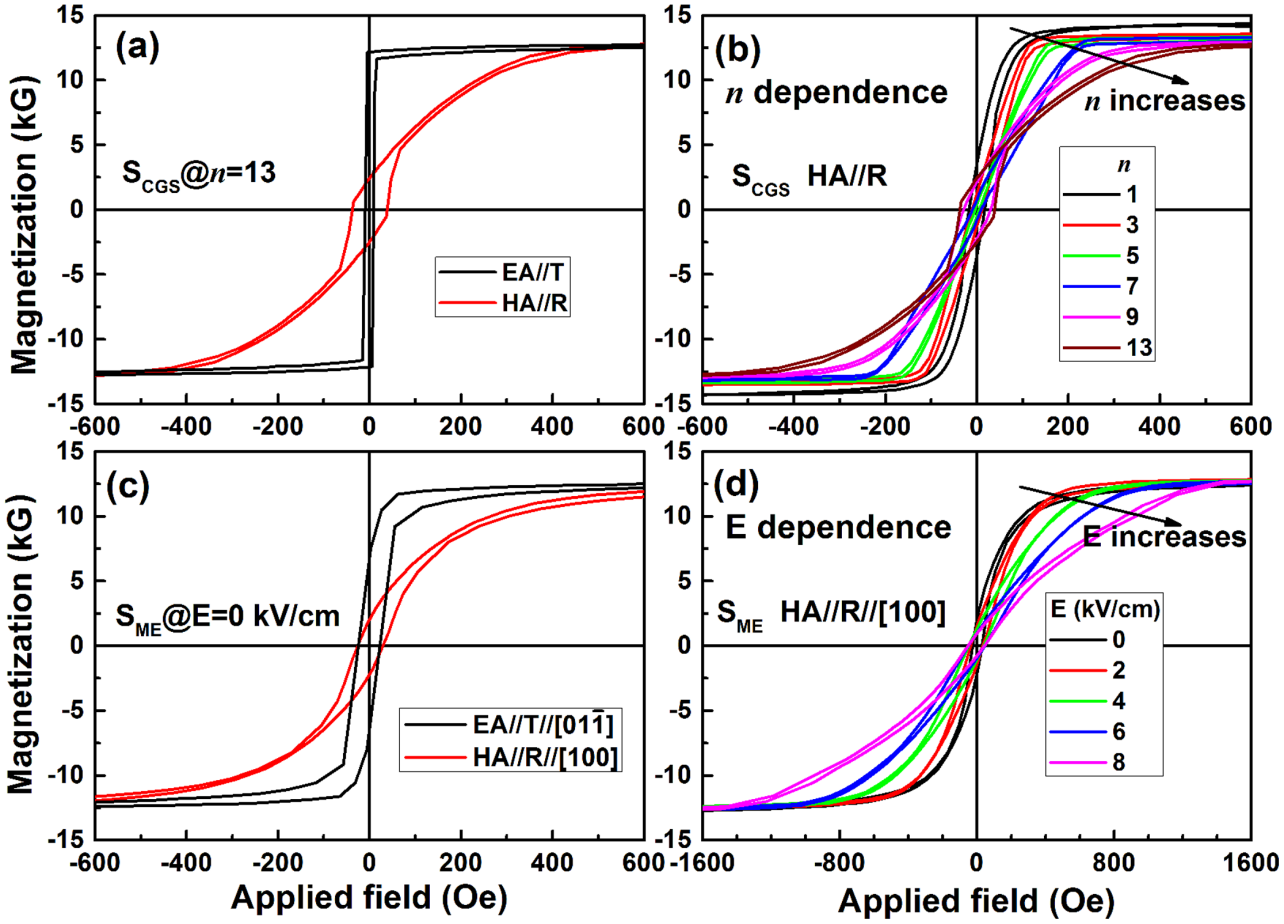
Sample position and electric field dependent hysteresis loops for S_CGS_ and S_ME_. (a) Representative hysteresis loops of S_CGS_@*n* = 13, showing an obvious uniaxial magnetization with HA along R direction; (b) the sample position *n* dependence of hysteresis loops along HA//R direction for S_CGS_; (c) the uniaxial magnetic hysteresis loops of S_ME_@E = 0 kVcm^−1^; (d) the electric field dependence of hysteresis loops of S_ME_.

Some typical hysteresis loops of S_CGS_ are summarized in Figure 4a and 4b. As illustrated in Figure 4a, there is an obvious uniaxial magnetic anisotropy with magnetically easy axis (EA) along tangential direction and magnetically hard axis (HA) along R direction for S_CGS_@*n* = 13. The *n*-dependent hysteresis loops along HA are shown in Figure 4b. It can be seen that the H_K_ increases with the increase of *n* (or B doping). The detailed H_K_ variation is shown in Figure 2. The uniaxial magnetic anisotropy in CGS films can be explained as stress gradient induced uniaxial magnetic anisotropy. The intrinsic stress in general is randomly dispersed in the magnetic films which gives rise to a high damping constant and decreasing resonant frequency. However, as reported in our previous work^28^,^29^,^30^, if a uniaxial stress replaces the randomly dispersed intrinsic stress, a stress-induced uniaxial magnetic anisotropy will be obtained. This uniaxial magnetic anisotropy leads to enhanced ferromagnetic resonance and improved microwave magnetic properties. The composition gradient sputtering is an effective way to induce a uniaxial stress, a high uniaxial magnetic anisotropy and an enhanced ferromagnetic resonance frequency.

### Combination of stress-mediated CGS and magnetoelectric coupling, and enhancement of microwave soft magnetic properties

As described above, the composition gradient sputtering method gives rise to a compressive stress, leading to a magnetically hard axis along the radial direction. On the other hand, for a (011)-cut PZN-PT single crystal substrate, when the electric field is applied along [011] direction, compressive and tensile stresses will be generated along [100] and [01-1] directions, respectively. According to magnetoelastic energy equation 
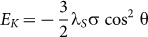
, for a positive *λ*_S_ (as the case in this study for the FeCoB films), a compressive or tensile stress σ will lead to a magnetic anisotropy that forces the magnetic moments to align perpendicular or parallel to the stresses direction, respectively. In other words, the magnetoelectric-coupling-induced magnetic hard axis direction is along [100] direction. So if the [100] direction is parallel to the R direction, the electric-field-tunable effective uniaxial magnetic anisotropy field (H_K_)_ME_ will be parallel to the composition-gradient-induced uniaxial magnetic anisotropy field (H_K_)_CGS_. Therefore they will be combined together leading to an ultrahigh and tunable uniaxial magnetic anisotropy field H_K_ (see the bottom of Figure 1a inset). In this study, sample position *n* = 13 was chosen to paste the PZN-PT substrate on, with substrate [100]//R to realize the superposition of two magnetic anisotropies. As expected, the hysteresis loops of CGS FeCoB/PZN-PT multiferroic heterostructures show a well-defined uniaxial magnetic anisotropy with a H_K_ of 384 Oe at E = 0 kVcm^−1^, slightly smaller than that of 402 Oe on Si substrate (see Figure 4a and 4c). It is exciting that the H_K_ of S_ME_ dramatically increases with the increase of electric field, and a record high H_K_ of 1498 Oe was obtained at E = 8 kVcm^−1^, implying that an ultrahigh ferromagnetic resonance frequency will be achieved thanks to the combining of composition gradient sputtering and magnetoelectric coupling effect.

**Figure 5:**
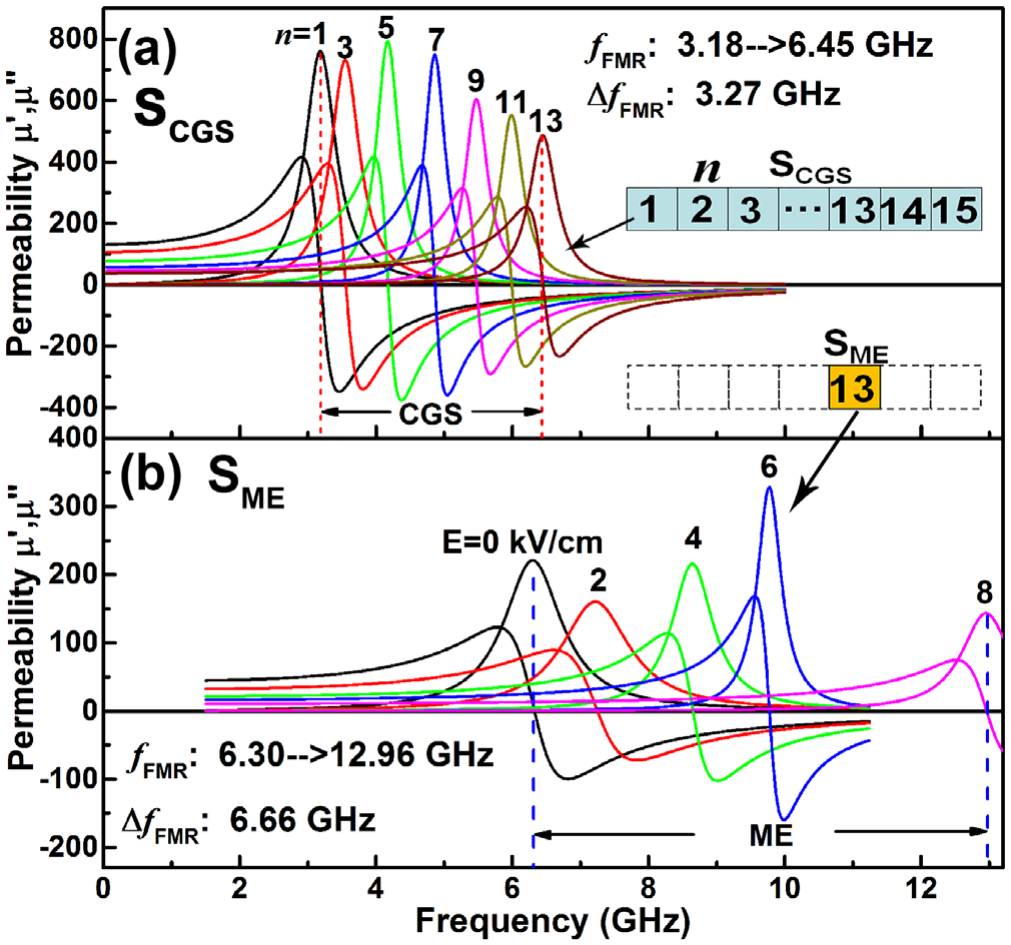
Frequency dependence of permeability at zero magnetic field for (a) S_CGS_ (*n* = 1–13) and (b) S_ME_ (E = 0–8 kVcm^−1^), showing the two-step climbing of *f*_FMR_ by CGS and ME effects, respectively.

Figure 5 shows the frequency dependence of complex permeability for S_CGS_ at various *n* and for S_ME_ at various electric fields. From Figure 5a, it can be seen that the CGS pushed the *f*_FMR_ from 3.18 to 6.45 GHz for *n* from 1 to 13. When the Si substrate was replaced by a PZN-PT substrate, it was found that although the *f*_FMR_ of 6.30 GHz for S_ME_@E = 0 kVcm^−1^ is slightly smaller than that of S_CGS_@*n* = 13 due to the difference between the Si and PZN-PT substrates, the magnetoelectric coupling effect dramatically drives the *f*_FMR_ of FeCoB/PZN-PT multiferroic heterostructures towards 12.92 GHz, directly reaching Ku-band from C-band across X-band. To the best of our knowledge, it is the first report that the ferromagnetic resonance frequency of as-deposited metallic magnetic films can reach Ku-band at zero-bias magnetic field. The magnetoelectric coupling effect in S_ME_ not only generates a 6.66 GHz shift of *f*_FMR_ under an electric field of 8 kVcm^−1^, but also provides an electric field tunable ferromagnetic resonance frequency shift over a very broad frequency span, realizing electric field controlled frequency tuning. This is of great significance because it provides the possibility to fabricate electric field tunable microwave devices with large tunability, low energy consumption and light-weight.

The electric field and composition gradient-induced ferromagnetic resonance frequency shift can be explained by the strain/stress-mediated in-plane magnetic anisotropy field. The in-plane ferromagnetic resonance frequency [Equation (1)] can be rewritten as:


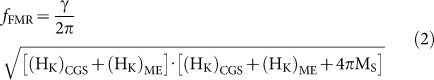
where (H_K_)_CGS_ is the CGS-induced uniaxial magnetic anisotropic field, (H_K_)_ME_ is the electric-field-induced effective magnetic field which could be positive or negative, and in this study it can be express as^35^,^36^,^37^,


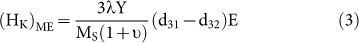
where Y is the Young's Modulus, ν is Poisson's ratio, λ is the magnetostriction constant of FeCoB film, d_31_ = −3000 pC N^−1^ along [100] and d_32_ = 1100 pC N^−1^ along [01-1] are linear anisotropic piezoelectric coefficients of PZN-PT, and E is the applied external electric field. (H_K_)_ME_ is quantitatively determined by observing the ferromagnetic resonance spectrum shift under various electric fields^38^. From equation (3), it can be concluded that the (H_K_)_ME_ is proportional to the applied electric field due to the magnetoelectric coupling effect, resulting in an electric field tunable *f*_FMR_. Similarly, the increase of (H_K_)_CGS_ due to the composition gradient will give rise to an upward shift of *f*_FMR_.

**Figure 6:**
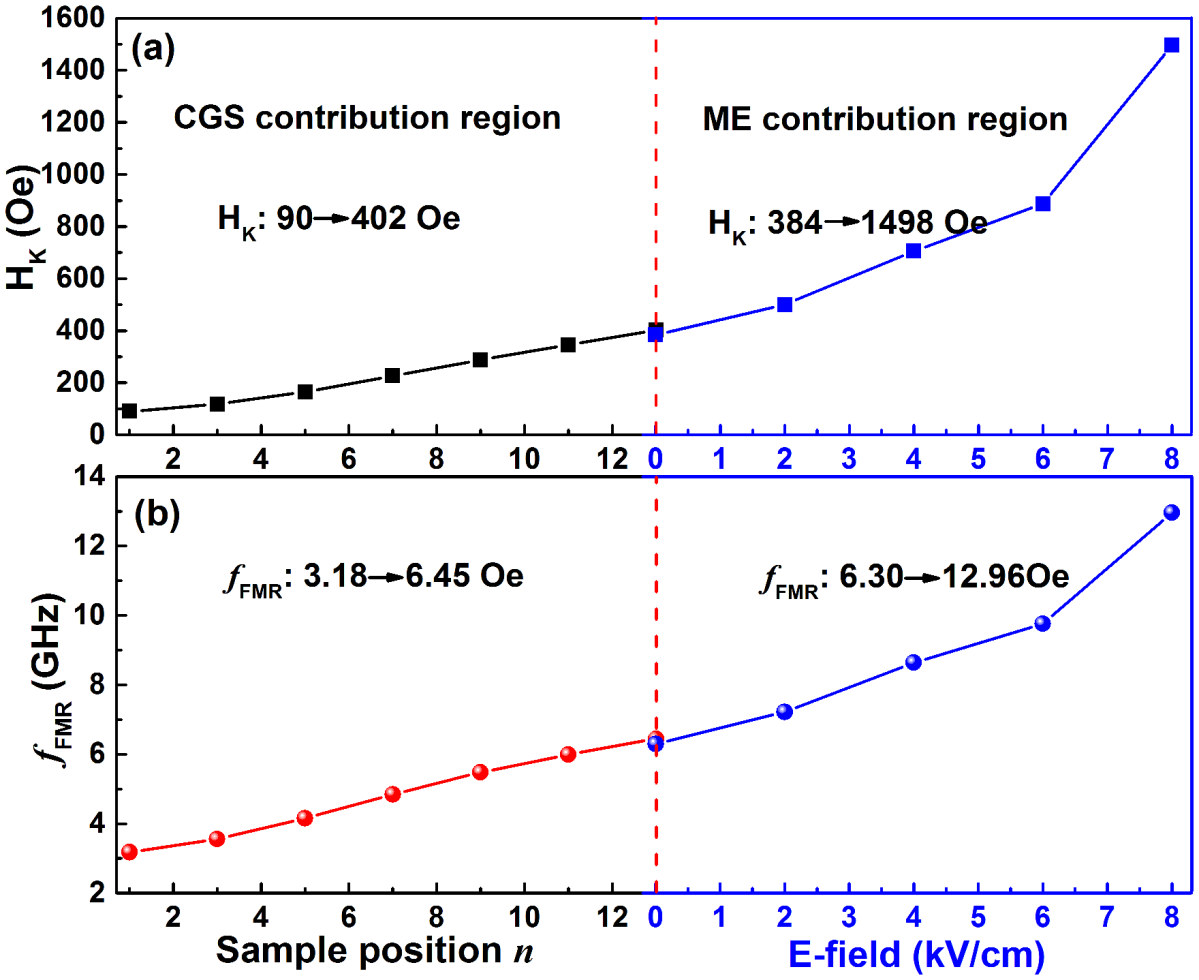
Sample position *n* and electric field dependence of (a) H_K_ and (b) *f*_FMR_ for S_CGS_ and S_ME_.

The sample position *n* and electric field dependence of magnetic anisotropy field H_K_ and ferromagnetic resonance frequency *f*_FMR_ are summarized in Figure 6. As illustrated, the two-step enhancement of H_K_ and *f*_FMR_ is clearly observed. Figure 6 is separated into left and right sections by a red dashed line. The left and right sections represent the contributions from composition gradient sputtering and magnetoelectric coupling effect, respectively. In the left section, with the increase of sample position *n*, the B concentration increases, and the H_K_ increases linearly from 90 to 402 Oe, leading to an increase of *f*_FMR_ from 3.18 to 6.45 GHz. In the right section, the CGS FeCoB film was deposited on PZN-PT substrate, and the enhancement effect of electric field on H_K_ and *f*_FMR_ begins to function in addition to the CGS effect. So the H_K_ and *f*_FMR_ are further pushed up by electric field starting from the corresponding values of S_CGS_. The H_K_ and *f*_FMR_ increase from 384 to 1498 Oe and from 6.30 to 12.96 GHz, respectively, with electric field from 0 to 8 kVcm^−1^. This two-step superposition method provides a combined effective uniaxial magnetic anisotropy field of up to 1498 Oe at 8 kVcm^−1^, which is 1–2 orders of magnitude higher than that by the conventional magnetic annealing method. At the same time, the *f*_FMR_ also directly reaches Ku-band from C-band across X-band.

It is worth mentioning that the strain occurs in the magnetic films due to the composition gradient and the magnetoelectric coupling effect, so a perpendicular anisotropy may arise^39^. For verifying this issue, the FMR measurement for S_ME_ sample was carried out at various electric fields. In the case of an in-plane applied magnetic field and measuring FMR along the easy axis, the in-plane resonance frequency is well described by Kittle equation, as reported in Ref. 39,40,41,


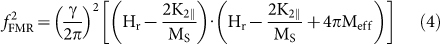
where 4πM_eff_ = 4πM_S_ – 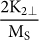 defines the effective saturation induction, 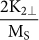 is the perpendicular anisotropy field H⊥, and 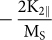 is the in-plane uniaxial anisotropy field H_eff_ ( = (H_K_)_CGS_ + (H_K_)_ME_). The 4πM_eff_ and H_eff_ can be evaluated by fitting the *f* vs. H_r_ plots using equation (4). The fitted H_eff_ is well consistent with the statically measured H_K_ indicating the experimental data are well fitted using Kittle equation. The FMR fitting results demonstrate that 4πM_eff_ with an average value of 12.37 kG is slightly smaller than 4πM_S_ of 12.79 kG with a small difference of 420 Oe, indicating that a small perpendicular anisotropy may present in the ferromagnetic film. Comparing with the out-of-plane saturation magnetic field of more than 15 kOe, such a small perpendicular field is not enough to drive the magnetic moments to the normal direction of the film. Therefore, the magnetic moments are mainly lying in the plane.

In conclusion, a record high ferromagnetic resonance frequency of 12.92 GHz, which directly reaches Ku-band from C-band across X-band, was obtained in as-deposited CGS FeCoB/PZN-PT multiferroic heterostructures at zero bias magnetic fields due to combining the composition gradient sputtering and magnetoelectric coupling effect together. This two-step superposition method can effectively add two kinds of uniaxial magnetic anisotropy fields together, obtaining ultrahigh H_K_ that cannot be reached with any single method. This method paves the way to get higher *f*_FMR_ by two-step or multi-step method. The CGS FeCoB/PZN-PT multiferroic heterostructures exhibit ultrahigh *f*_FMR_ with very broad electric field tunable frequencies, which provides great opportunities for self-biased voltage tuning microwave multiferroic components working at X-band or higher frequencies without energy consuming electromagnets. All the fabrication processes of the CGS FeCoB/PZN-PT multiferroic heterostructures are carried out at room temperature (i.e. at IC compatible process), which is very beneficial to the integration of these soft magnetic films into monolithic microwave integrated circuits.

## Methods

### Preparation of the FeCoB/PZN-PT multiferroic hyterestructures

The FeCoB films with average thickness of 100-nm were deposited on (100) single crystal Si substrates with dimension of 75 mm × 5 mm × 0.5 mm by composition gradient sputtering method at room temperature under 2.8 mTorr Ar atmosphere with a flow rate of 20 sccm, along with a RF power of 80 W for Fe_70_Co_30_ target and various powers from 60 to 180 W for B target. The FeCoB films deposited on Si substrate were used to measure magnetic properties, to observe microstructure, and to explore optimum deposition condition. It is found that the sputtering powers of 80 W for Fe_70_Co_30_ and 135 W for B target are the optimum deposition condition. Based on the exploring data with Si substrate (as discussed above), the optimum position is at *n* = 13. Afterwards, a (011)-cut single crystal PZN-PT substrate with dimension of 5 mm[100] × 5 mm[01-1] × 0.5 mm[011] was pasted at position *n* = 13 (see Figure 1 inset) with [100]//R, and deposited CGS FeCoB film on it under the optimum deposition condition.

### Measurement of composition, magnetic, and microwave properties

The composition of films was determined by a FE-EPMA. The magnetic properties were measured by a vibrating sample magnetometer (VSM). The ferromagnetic resonance characteristics of the multiferroic heterostructures were analyzed by a broadband ferromagnetic resonance spectroscopy with the transmission line along the easy axis. The microwave performances were evaluated a vector network analyzer (VNA) with co-planar waveguide. The vector network analyzer acts as a transmitter and a receiver of microwave. The film sample was put on a specially designed co-plane waveguide transmission line fixture. When the microwave passes through the transmission line covered with the soft magnetic film, it will be absorbed by the magnetic film. As a result, the scattering parameter S_21_ will show an absorption peak around the ferromagnetic resonance frequency. The vector network analyzer records the scattering parameters, and simulates the measured curves with LLG (Landau-Liftshitz-Gilbert) equation. Thus, useful parameters such as permeability, ferromagnetic resonance frequency, damping constant, etc. can be obtained.
